# Study on Manufacturing Technology of Ag-8.5Au-3.5Pd Fine Alloy Wire

**DOI:** 10.3390/mi12080938

**Published:** 2021-08-09

**Authors:** Jun Cao, Junchao Zhang, Baoan Wu, Huiyi Tang, Changchun Lv, Kexing Song, Guannan Yang, Chengqiang Cui, Yangguang Gao

**Affiliations:** 1 School of Mechanical and Power Engineering, Henan Polytechnic University, Jiaozuo 454000, China; zjc_xlyx@sina.com; 2 Chongqing Materials Research Institute Co., Ltd., Chongqing 400700, China; wubaoan@163.com (B.W.); hytang320@163.com (H.T.); 3 Henan Youk Electronic Material Co., Ltd., Jiyuan 454650, China; ykdz0391@163.com (C.L.); caolinc@163.com (Y.G.); 4 School of Materials Science and Engineering, Henan University of Science and Technology, Luoyang 471000, China; kxsong@haust.edu.cn; 5 State Key Laboratory of Precision Electronic Manufacturing Technology and Equipment, Guangdong University of Technology, Guangzhou 510006, China; ygn@gdut.edu.cn (G.Y.); cqcui@gdut.edu.cn (C.C.)

**Keywords:** alloy wire, processing rate, annealing, strength, elongation, resistivity, tension

## Abstract

The performance of Ag-8.5Au-3.5Pd alloy wire after cold deformation and annealing were analyzed by SEM (scanning electron microscope), strength tester and resistivity tester. The processing process and performance change characteristics of Ag-8.5Au-3.5Pd alloy wire were studied. The results show that alloy wire grains gradually form a fibrous structure along with the increase in deformation. The strength of the wire increases with the increase in deformation rate, but the increase trend becomes flat once the deformation rate is higher than 92.78%; the resistivity of Ag-8.5Au-3.5Pd alloy wire decreases with the increase in annealing temperature, reaching minimum (2.395 × 10^−^^8^ Ω·m) when the annealing temperature is 500 °C; the strength of Ag-8.5Au-3.5Pd alloy wire decreases with the increase in annealing temperature. When the annealing temperature is 500 °C, the strength and elongation of the φ0.2070 mm Ag-8.5Au-3.5Pd alloy wire are 287 MPa and 25.7%, respectively; the fracture force and elongation of φ0.020 mm Ag-8.5Au-3.5Pd alloy wire are 0.0876 N and 14.8%, respectively. When the annealing temperature is 550 °C, the metal grains begin to grow and the mechanical performance decrease; the φ0.020 mm Ag-8.5Au-3.5Pd alloy wire have good surface quality when the tension range is 2.5–3.0 g.

## 1. Introduction

With the development of semiconductor devices and integrated circuits toward multilead packaging, high integration and miniaturization, more attention has been paid to ultra-fine, low-cost, high-temperature-resistant wires [1,2,3]. Bonding gold wires are affected by the shortcomings of high-cost, low strength and brittle intermetallics and are gradually to be replaced by other materials [4,5]. The cost of copper wire is low, but it still has some drawbacks such as easy oxidation and high hardness [6,7,8]. Aluminum wires have weaker tensile strength and heat resistance than gold wires, so they are prone to collapse and droop [9]. Nowadays, there are many kinds of bonding materials, among which the silver base alloy bonding wires exhibit excellent mechanical properties, good oxidation resistance and high reliability, and are cost effective. As a result, they can limit light attenuation and improve the conversion rate in light-emitting diode (LED) packaging. Because of these advantages, these wires are employed widely in integrated circuit and LED packaging [10,11,12,13,14,15,16,17]. However, for pure Ag wire, due to high thermal conductivity and low strength of high temperature, the parameter range is small during the bonding process, and has high failure probability of ball bonding points under high temperature conditions, thus reducing production efficiency and service life of high-power LED devices [18,19,20,21,22,23]. High-performance silver-based alloy wires obtained by alloying is an effective method to improve the performance of silver wire. Au (gold) and Pd (palladium) elements have similar performance to Ag and can be infinitely soluble with each other. The addition of Au and Pd elements can improve the strength and high temperature stability of silver wire [24], and increase the parameter window range and interface bonding strength during bonding process [25], and inhibit the growth of intermetallics at interface (especially under high temperature and humidity conditions), which is conducive to further increasing interface reliability [26] and enhancing device life. The research and development of silver-based alloy wire with Au and Pd solves many problems in the application of single-element wire such as gold wire, copper wire and silver wire, and have broad application prospects in chip packaging such as high-density, large-scale integrated circuits and high-power LED. In recent years, many scholars have carried out a lot of research on Ag/Au/Pd alloy wires. Feng, D. et al. [27] studied the thermodynamic performance of Ag-Au-Pd alloy by electrochemical methods and concluded that the thermodynamic stability of Ag-Au-Pd is significantly higher than Ag. Guo, R. et al. [28] studied the intermetallics at the interface of Ag-8Au-3Pd alloy wire and concluded that AuAl_2_^+^ (Au, Ag) 4Al and Ag_2_Al intermetallics were generated at the interface of Ag-8Au-3Pd/Al, and the Ag_2_Al intermetallics layer could effectively prevent the diffusion of Au atoms to the interface of Al. Cao, J. et al. [29,30] studied the cold deformation and annealing process of Ag-4Pd alloy wire and summarized the influence of the performance and structure of Ag-4Pd alloy on strength. It is concluded that twin crystal structure appears during the annealing of the Ag-4Pd alloy wire, and twin nucleation and subcrystalline annexation to large nucleation is the main nucleation mode, and the length of the heat-affected zone is shorter than Ag wire. Most of them involve the research on the reliability of Ag/Au/Pd alloy wire and the Ag/Al interface, while there is little discussion on wire performance and processing technology of Ag/Au/Pd alloys. In this paper, the influence of the deformation rate and annealing on the performance of the Ag-8.5Au-3.5Pd alloy wire during processing is studied, and the processing technology of the Ag-8.5Au-3.5Pd alloy wire is further explored, which provides a theoretical basis for the manufacture of Ag-8.5Au-3.5Pd alloy wire.

## 2. Test Materials and Methods

### 2.1. Test Materials

φ8 mm diameter Ag-8.5Au-3.5Pd alloy rod and drawing dies with a diameter range of 8.00–0.020 mm.

### 2.2. Test Method

**Figure 1:**
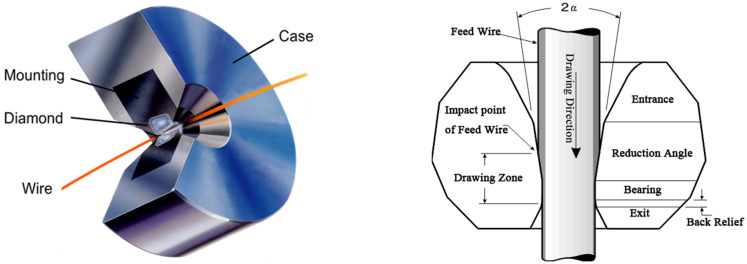
Drawing die structure.

The φ8 mm Ag-8.5Au-3.5Pd melting cast alloy rod was cold deformed by large drawing, medium drawing, fine drawing and ultra-fine drawing. The structure, outline size and hole size of drawing dies are shown in Figure 1, Table 1 and Table 2, respectively. Firstly, the alloy wire is machined to φ1.1008 mm on the single-die drawing machine, and the compression rate and drawing speed of the alloy wire are 10% and 20%, 10 m/min and 30 m/min in the range of dies with a hole diameter of 8.00–5.2488 mm and 4.6947–1.1008 mm, respectively. The drawing solution is 396V2 water-soluble solution and the concentration is 20% (the composition of the drawing solution is 50% polyethylene glycol, 30% dehydrated sorbitol monooleate polyoxyethylene ether and 20% water). A 10-times magnifying glass is used to inspect the surface of the wire at any time during rough drawing, and the surface is required to be smooth. Then, the φ1.1008 mm alloy wire is machined to φ0.2070 mm on the LH160 Mid-Drawing wire drawing machine, and the compression rate and drawing speed of the alloy wire is 13% and 200 m/min. In this process, the drawing solution is water-soluble, and the concentration is 5% and the temperature is 40 °C. The φ0.2070 mm alloy wire is heat treated according to the temperature and time shown in Table 3, and the fluctuation range of the annealing temperature is ±2 °C. The annealing tube is made of quartz glass and the length is 2000 mm. The tension of the wire is controlled by angular displacement sensor in the annealing equipment and the tension range is 1.0–10.0 g. High purity N_2_ protection is used in the annealing process. In the process of rough drawing, both the alloy wires of different diameters (φ8.0 mm, φ5.2488 mm, φ3.3592 mm, φ2.1499 mm, φ1.1008 mm, φ0.2070 mm) after cold deformation and the φ0.2070 mm alloy wire after annealing are sampled. The alloy samples were corroded for 3~5 s by the solution (30% mass fraction hydrogen peroxide and 20% mass fraction ammonia mixed in the same proportion). The solution should be used immediately once prepared. The microstructure morphology and grain size of the alloy wire under different deformation variables were observed by JEOL JSM-6700F SEM and ZEISS SEM. Finally, mechanical and electrical performance of Ag-8.5Au-3.5Pd alloy wires that annealed at different temperatures were tested on the KDDII-0.01 tension machine and ZX01 double-arm electric bridge, respectively, and the effects of different deformation amounts and annealing temperatures on the wire performance were studied. The tensile test samples are 100 mm in length and 10 mm/min in tension speed. The electrical performance test sample length is 1000 mm; the resistance value is measured by 4 probe method with the ZDCY-80 intelligent resistance tester and then according to the formula ρ = R × S/L calculates the resistivity of the alloy wire (ρ is resistivity, L is wire length, S is section area).

The φ0.2070 mm Ag-8.5Au-3.5Pd alloy wire treated by optimized annealing conditions was cold-worked on the SPS-100 drawing machine. The drawing process includes two passes, and the diameter ranges of the dies are φ0.2070–φ0.1158 mm and φ0.11169–φ0.0648 mm in the first and second pass, respectively; the compression rate is 7%, and the drawing speed is 500–600 m/min. The drawing solution is water-soluble, which concentration is 2.0% and the temperature is 40 °C. Then, the φ0.06481 mm alloy wire is drawn by the MK-30 no-sliding wire drawing machine. The drawing process also includes two passes. In the first pass, the rang of the die diameter is φ0.06250–φ0.03761 mm and the compression rate is 7%. In the second pass, the range of the die diameter is φ0.03636–φ0.02513 mm and the compression rate is 6.5%. The drawing speed is 500–600 m/min. The drawing solution is 396V2 water-soluble solution which concentration is 0.5% and the temperature is 40 °C. Finally, the φ0.02513 mm alloy wire is processed to φ0.01962 mm on the WSS-11 Micro wire drawing machine; the compression rate is 6.0% and the drawing speed is 300–400 m/min. The drawing solution concentration is 0.5% and the temperature is 40 °C.

## 3. Results

### 3.1. Analysis of the Influence of Cold Deformation on the Microstructure of Ag-8.5Au-3.5Pd Alloy Wire

**Figure 2:**
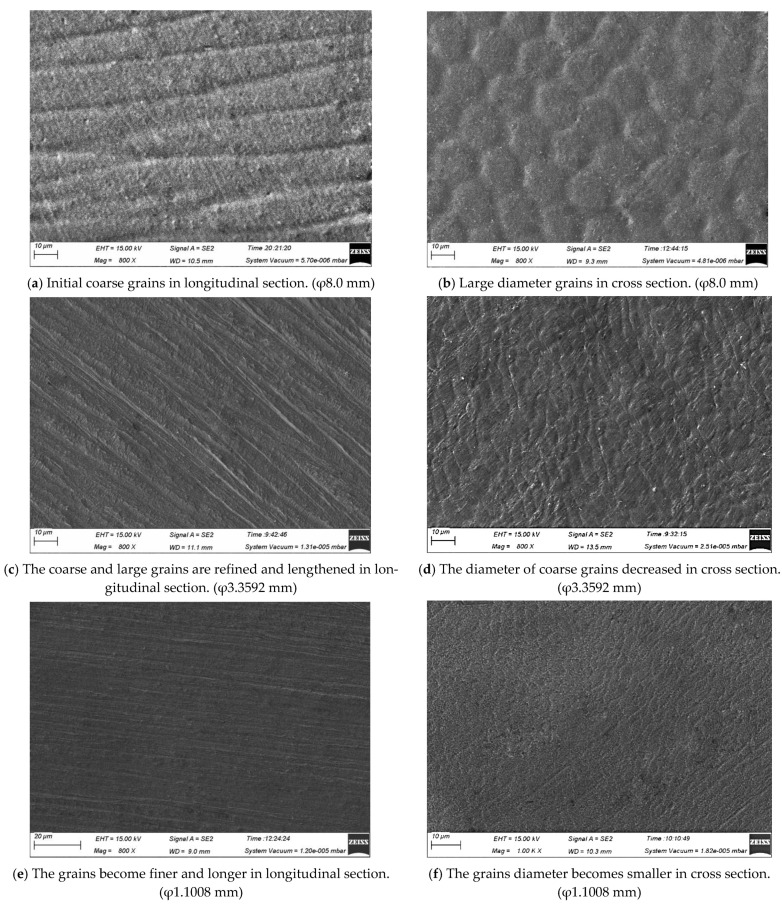

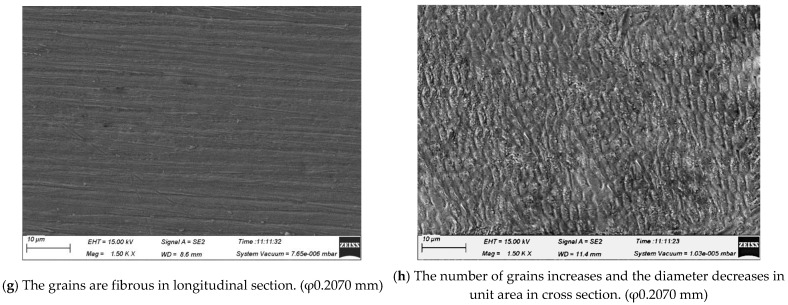
Microstructure and morphology of Ag-8.5Au-3.5Pd alloy wires with different deformation variables.

Figure 2 shows the microstructures in different deformation (φ8.0 mm, φ5.2488 mm, φ3.3592 mm, φ2.1499 mm, φ1.1008 mm and φ0.2070 mm) of the Ag-8.5Au-3.5Pd alloy wire after multi-pass drawing. It can be seen that during plastic deformation, the grains are gradually straightened and refined, and finally the microstructures present fibrous (as shown in the microstructure of φ0.02070 mm size) with the increase in deformation of the wire. In addition, the number of grains and the degree of microstructure distortion also increase.

### 3.2. Effect of Cold Deformation on Performance of Ag-8.5Au-3.5Pd Alloy Wire

**Figure 3:**
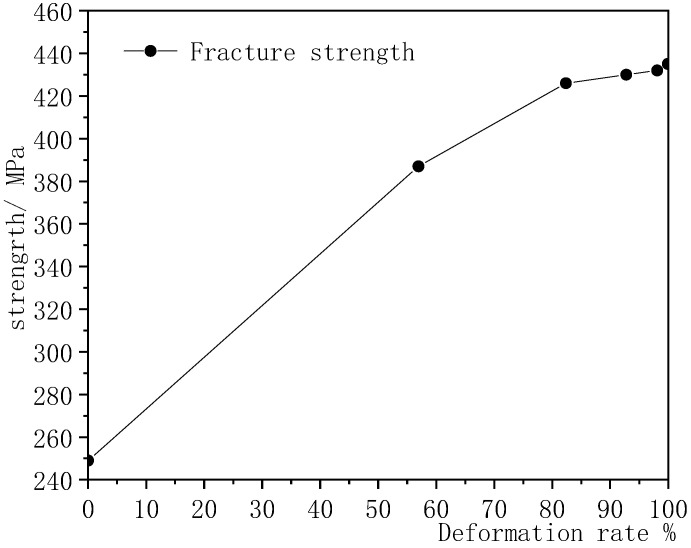
Fracture strength of Ag-8.5Au-3.5Pd alloy wires with different deformation rates.

The curve of mechanical performance of Ag-8.5Au-3.5Pd alloy wires with different deformation values (φ8.0 mm, φ5.2488 mm, φ3.3592 mm, φ2.1499 mm, φ1.1008 mm, φ0.2070 mm) after multi-pass drew are shown in Figure 3 (deformation rates are 0%, 56.95%, 82.37%, 92.78%, 98.11% and 99.93%, respectively). It can be seen that the fracture strength of the wire increases rapidly with the increase in the deformation rate. That is because the alloy wires are processed by cold deformation; the internal grains become finer and smaller and the number of grains increases, as well as the dislocation increasing. However, when cold deformation is processed to a certain extent, the effect of cold deformation on grain refinement is weakened, so when the deformation rate is higher than 92.78%, the increase trend of the strength of the Ag-8.5Au-3.5Pd alloy wire becomes smooth and steady.

### 3.3. Effect of Different Annealing Temperatures on Electrical Performance of Ag-8.5Au-3.5Pd Alloy Wire

**Figure 4:**
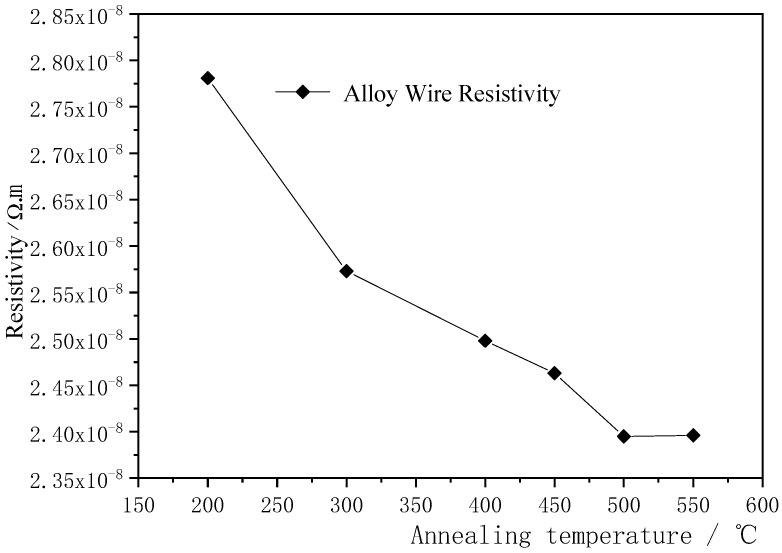
Changes of resistivity of Ag-8.5Au-3.5Pd alloy wire with different annealing temperatures.

The curve of the influence at different annealing temperatures on the wire resistivity of φ0.2070 mm Ag-8.5Au-3.5Pd alloy wire is shown in Figure 4. The annealing time is 2.4 s. In this figure, the resistivity decreases from 2.781 × 10^−^^8^ Ω∙m down to 2.395 × 10^−^^8^ Ω∙m with the increase in annealing temperature, and the minimum is 2.395 × 10^−^^8^ Ω∙m when the annealing temperature is 500 °C. The resistivity of the alloy wire almost remains unchanged even if the temperature continues to increase.

The resistivity of metal is mainly caused by phonon, dislocation, point defects (soluble atoms, impurities and vacancies, etc.) and the scattering effect of the interface on electrons [31]. As the increase in the annealing temperature of alloy wires which have been cold worked, the resistivity components change and the corresponding conductivity changes as well. During annealing, some solute atoms gradually precipitate from the matrix under a little free energy released, resulting in a decrease in concentration. Lattice distortion caused by atomic radius difference becomes effectively mitigated and tends towards orderly and periodic arrangement [32,33]. At the same time, the diameter and spacing of the fibers are reduced due to the refinement of the microstructure. The fine fiber structure cannot contain more dislocation substructures, which results in the absorption of dislocations by the grains interface so that the dislocation density decreases. The scattering effect of free electrons during their movement decreased, thus reducing the resistivity of Ag-8.5Au-3.5Pd alloy wires.

### 3.4. Effect of Different Annealing Temperatures on the Mechanical Performance of Ag-8.5Au-3.5Pd Alloy Wire

**Figure 5:**
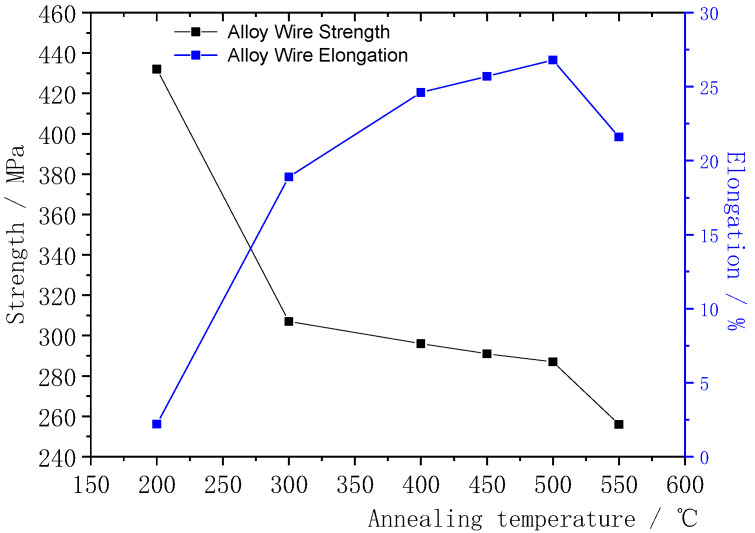
Changes of strength and elongation of Ag-8.5Au-3.5Pd alloy wire with different annealing temperatures.

The influence of different annealing temperatures on the mechanical performance of φ0.2070 mm Ag-8.5Au-3.5Pd alloy wire is shown in Figure 5. It can be seen that the alloy wire begins to recover when the annealing temperature is 300 °C, which eliminates the working-hardening caused by cold working. Its strength decreased significantly from 428 MPa (200 °C) to 307 MPa, and the decrease rate was 28.3%. The elongation increased from 2.2 to 18.5% and the increase rate was 88.1%, and the deformed structure almost all disappeared. When the annealing temperature was 500 °C, the alloy wire fully recovered and began to recrystallize; the grain fiber structure of the alloy wire changed, and the strength decreased to 287 MPa and the elongation increased to 25.7%. As the annealing temperature increased to 550 °C, the grains began to grow, while the strength and elongation decreased to 256 MPa and 20.6%, respectively. It can be seen that the alloy wire has good mechanical performance when the annealing temperature is 500 °C.

### 3.5. Effect of Different Annealing Parameters on Microstructure and Morphology of Ag-8.5Au-3.5Pd Alloy Wire

**Figure 6:**
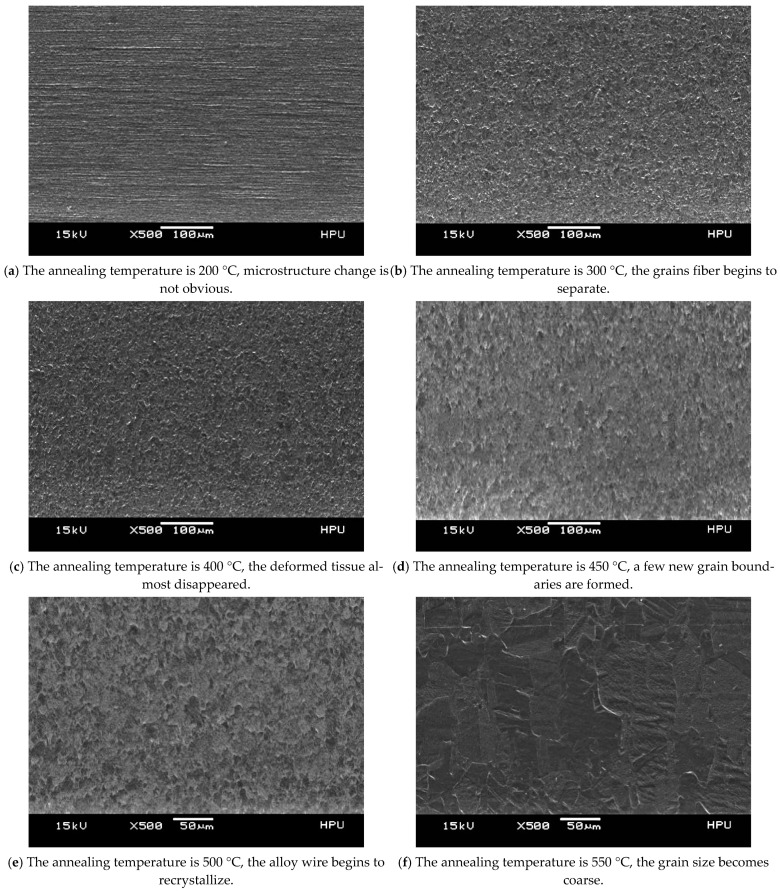
Microstructure and morphology of Ag-8.5Au-3.5Pd alloy wire at different annealing temperatures.

The microstructure morphology changes of φ0.2070 mm Ag-8.5Au-3.5Pd alloy wire after cold working deformation at different annealing temperatures are shown in Figure 6. When the annealing temperature is 200 °C, only point defects and line defect movements exist in the structure, the lateral displacement of the boundaries of the fiber grain is easy and the interface of the grain’s fiber is slightly clear; there is no obvious change in the microstructure in Figure 6a. The point defect and dislocation migrate violently during annealing at 300 °C and 400 °C; the grain’s fiber is further separated and coarsened and the deformation structure basically disappears, but most of the dislocation migration is confined to the fiber scale. The fiber shapes observed by SEM are shown in Figure 6b,c. When the annealing temperature is 450 °C, the release tendency of deformation storage energy is increased and significant changes in grain shape are caused by the substantial reduction of crystal defects. A few new large angle grain boundaries are gradually formed, and a few recrystallized grains are produced in the grain’s fiber structure. The new nuclei are generally located in the dislocation substructure with a high density, and the deformation structure completely disappeared at this time. The alloy wire is at the initial stage of recrystallization, as shown in Figure 6d. The alloy wire begins to recrystallize when the annealing temperature is 500 °C, as shown in Figure 6e. The grain’s fiber is further coarsened locally and evolved into short bar or equiaxed fine grains distributed along the direction of the original fiber. The grains’ interface migration is thoroughly activated due to the increase in recrystallized grains and the increase of grain size. In order to reduce the total interfacial energy, the grain boundary migration starts to break through the limit of original grains boundary, which caused obvious distortion and local shrinkage of the grains interface; the fibers are even partially broken and separated, which leads to the gradual disappearance of composite fibers. In Figure 6f, when the annealing temperature was 550 °C, the grains began to grow bigger.

### 3.6. Effect of Wire Drawing on Fine Ag-8.5Au-3.5Pd Alloy Wire

**Figure 7:**
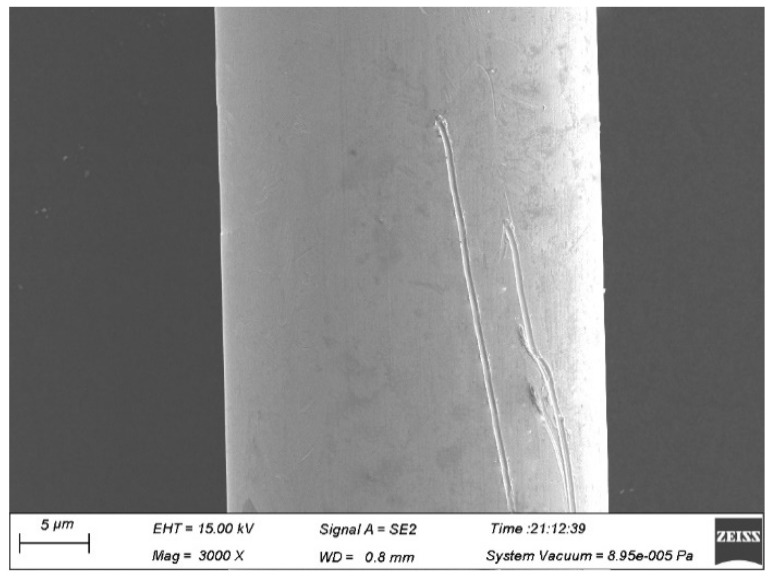
Scratches on the surface of Ag-8.5Au-3.5Pd alloy wire due to dirty guide wheel (0.020 mm).

The guide wheel quality has a great influence on the surface of the Ag-8.5Au-3.5Pd alloy wire in the fine drawing process. The surface damage can be caused by the inflexible rotation of the guide wheel or surface defect of the guide wheel during wire drawing, as shown in Figure 7. During the drawing process, stress will be concentrated on the damage or scratch on the surface of the alloy wire, which can easily cause serious distortion or fracture, while the contamination on the surface increases the pulling tension during the drawing process, resulting in uneven size of the alloy wire diameter.

The concentration of lubricant has a serious influence on the wire drawing process during the fine machining of the Ag-8.5Au-3.5Pd alloy wire. Low concentration solution will lead to poor lubrication, increase wear of the die and cause scratches, grooves and other defects on the surface of the alloy wire, while high concentration solution will block the hole and increase the drawing force. When the lubricant concentration is too high, the lubricant will stay in the lubrication zone of the die, which prevents the lubricant from entering the sizing and compression zones, so that the lubricant cannot play a lubricating role in processing. This causes the pulling force to increase and the wire to become finer, even causing defects and lubricant residue on the wire surface. Residual lubricant on the wire surface will cause serious contamination and accelerated wear of the dies. Water-soluble lubricant is used in the drawing process of alloy wire and the concentration of lubricant is 0.5%. The dilution of lubricant uses deionized water, because common tap water contains a lot of calcium ions, which are easy to alkalize and difficult to clean. This will have a negative impact on the surface of the alloy wire.

**Figure 8:**
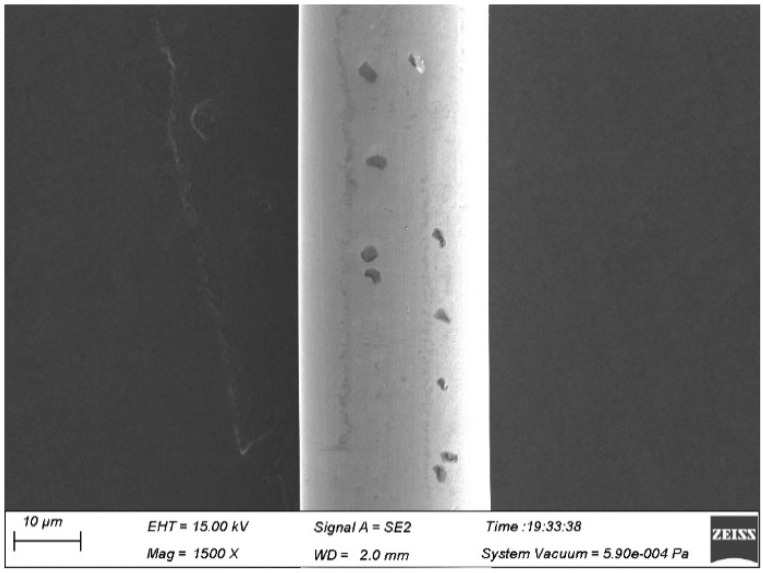
Surface contamination of Ag-8.5Au-3.5Pd alloy wire due to dirty guide wheel (0.020 mm).

**Figure 9:**
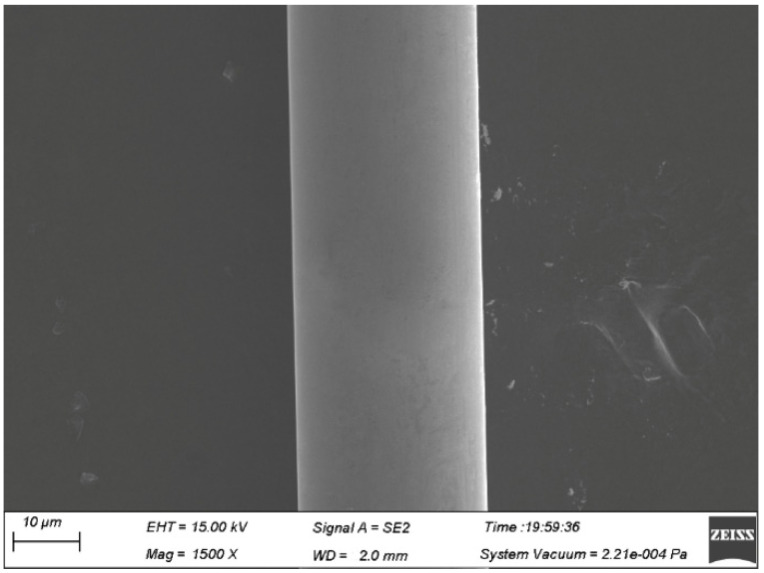
Perfect Surface of Ag-8.5Au-3.5Pd alloy wire (φ0.025 mm).

The surface quality of the Ag-8.5Au-3.5Pd alloy wire is highly required. If contamination exists on the wire surface (as shown in Figure 8), the bonding strength will be reduced, especially during the second bonding point process. Therefore, the surface of Ag-8.5Au-3.5Pd alloy wire needs to be cleaned after fine wire drawing. The cleaning method is on-line ultrasonic cleaning. Figure 9 shows the high-quality surface of Ag-8.5Au-3.5Pd alloy wire after cleaning.

### 3.7. Effect of Annealing Temperature on Mechanical Performance of Fine Ag-8.5Au-3.5Pd Alloy Wire

**Figure 10:**
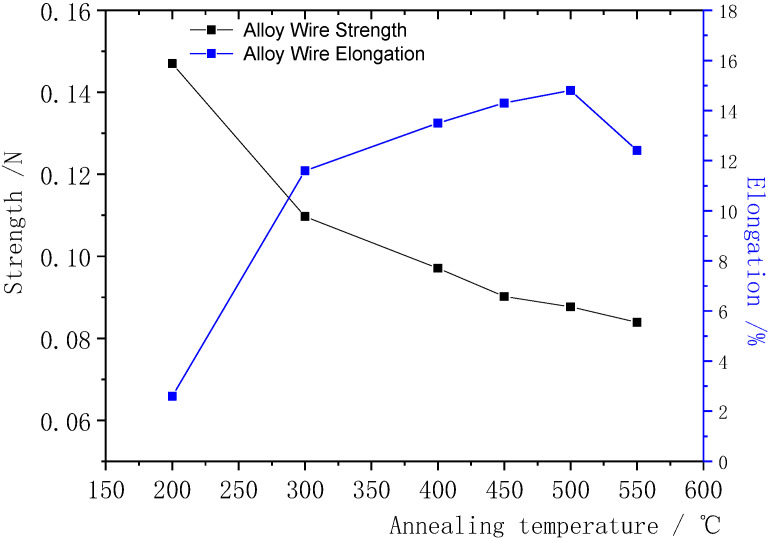
Relationship between annealing temperature and fracture strength and elongation of Ag-8.5Au-3.5Pd alloy wires.

The φ0.020 mm Ag-8.5Au-3.5Pd alloy wire was heat treated at different temperatures (the annealing time and speed were 0.8 s and 90 m/min, respectively). The influence of different annealing temperatures on the fracture force and elongation of the alloy wire was studied, and the results are shown in Figure 10.

**Figure 11:**
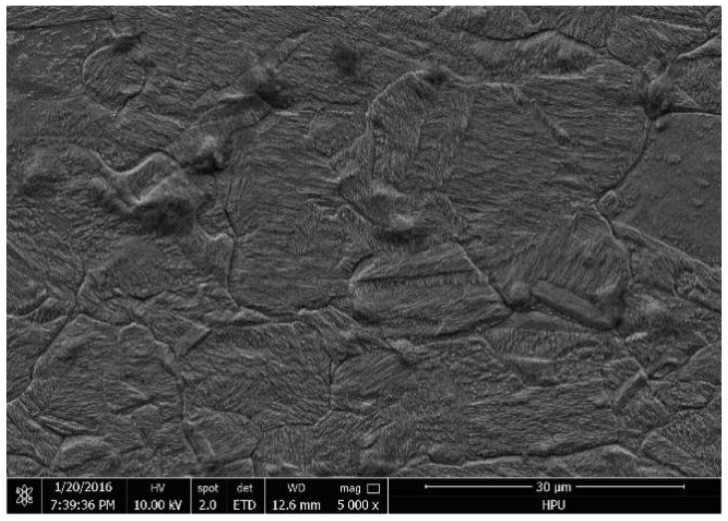
Coarse grains structural of Ag-8.5Au-3.5Pd alloy wire at 550 °C annealing temperature.

It can be seen that recovery occurred in the Ag-8.5Au-3.5Pd alloy wire with the increase in the annealing temperature, and eliminated the work hardening caused by the cold working; its tensile fracture force reduced while elongation increased. When the annealing temperature increased to 300 °C, the degree of recovery was further strengthened, the fracture force decreased smoothly to 0.1097 N and the elongation increased to 11.6%. The fracture force of the alloy wire decreased rapidly to 0.0971 N while the elongation increased to 13.5%, with the annealing temperature increasing to 400 °C. The recovery process is completed at this stage. When the annealing temperature was 450 °C, the fracture force of the Ag-8.5Au-3.5Pd alloy wire decreased to 0.0902 N; at the same time, the elongation increased to 14.3%. When the annealing temperature was 500 °C, the fracture force was 0.0877 N and the elongation was 14.8%; the alloy wire has excellent mechanical performance in this case. The Ag-8.5Au-3.5Pd alloy wire begins to recrystallize and produces coarse grains with further increase in the annealing temperature (550 °C), as shown in Figure 11. Coarse grains reduce the hindrance to dislocation movement, so that the alloy wire is more prone to stress concentration when subjected to tension, which results in a decrease in mechanical performance. The tensile force decreased to 0.0839 N and the elongation decreased to 12.4%. It can be seen from Figure 10 that the Ag-8.5Au-3.5Pd alloy wire has excellent mechanical performance when the annealing temperature is 500 °C.

### 3.8. Effect of Annealing Tension on Surface Quality of Ag-8.5Au-3.5Pd Alloy Wire

The φ0.020 mm Ag-8.5Au-3.5Pd alloy wire was heat treated in different tension values (1.5 g, 2.0 g, 2.5 g, 3.0 g, 3.5 g). The annealing time, speed and temperature are 0.8 s, 90 m/min and 500 °C, respectively.

**Figure 12:**
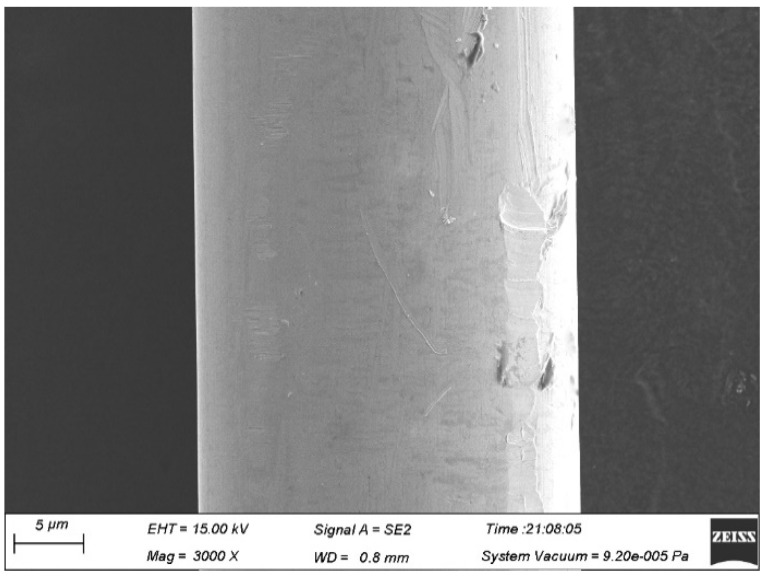
Surface mechanical damage of Ag-8.5Au-3.5Pd alloy wire caused by small tension (1.5 g) after anneal.

**Figure 13:**
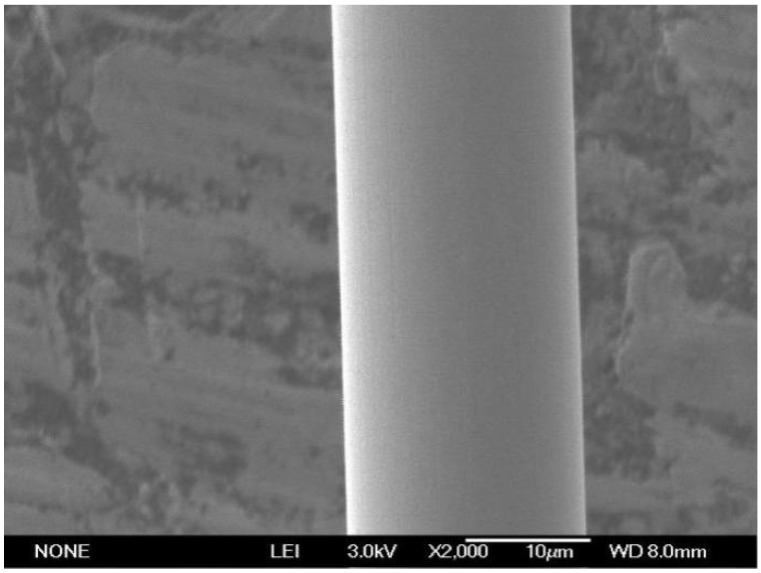
Good surface morphology of Ag-8.5Au-3.5Pd alloy wire after annealing (tension 2.0 g or 3.0 g).

**Figure 14:**
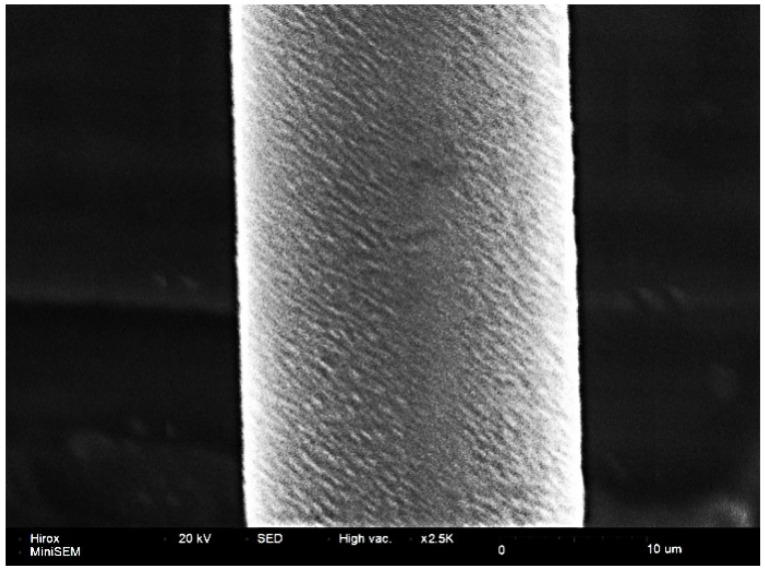
Surface corrugation defects of Ag-8.5Au-3.5Pd alloy wires due to excessive tension (3.5 g) during annealing.

When the annealing tension is small (1.5 g, 2.0 g), the alloy wire will vibrate seriously in the annealing tube, which results in contact with the annealing tube wall. The surface of the alloy wire is burned (as shown in Figure 12) because the wall temperature of the annealing tube is higher than the center temperature. When the annealing tension is 2.5 g or 3.0 g, the surface of the alloy wire is smooth and has no damage, as shown in Figure 13. The dynamic recovery and recrystallizing of the Ag-8.5Au-3.5Pd alloy wire will occur at a high annealing temperature. When the annealing tension is 3.5 g, because the tension acting on the wire is large at this time, the grains will slip under the tension and form corrugated defects on the surface of the wire, as shown in Figure 14. This defect will reduce the strength of the second bonded point, and thus reduce the reliability of the device.

In addition, the Ag-8.5Au-3.5Pd alloy wire will drawn finer in the annealing tube due to excessive tension (3.5 g). After annealing, the diameter of the φ 0.020 mm Ag-8.5Au-3.5Pd alloy wire becomes φ 0.0188 mm and the wire diameter is reduced by 6%, which cannot meet the requirements of industrialization. Therefore, the tension should be in the range of 2.5–3.0 g when the φ0.020 mm Ag-8.5Au-3.5Pd alloy wire is annealing.

## 4. Conclusions

(1) When Ag-8.5Au-3.5Pd alloy wire is plastic-deformed, metallic grains are gradually straightened and refined and finally show the fibrous structure. With the increase in deformation rate, the strength of the alloy wire increases, and the increase tends to be flat when the deformation rate is higher than 92.78%.
(2) The resistance value of Ag-8.5Au-3.5Pd alloy wire decreases from 2.781 × 10^−8^ Ω·m to the minimum 2.395 × 10^−8^ Ω·m (500 °C) with increasing annealing temperature, and it remains constant even if the temperature continues to rise.
(3) With the increase of annealing temperature, the Ag-8.5Au-3.5Pd alloy wire recovers and eliminates the hardening which was caused by cold working, and its strength is significantly reduced. When the annealing temperature is 500 °C, the alloy wire has good mechanical performance, while the grain size begins to grow and its mechanical performance decreases once the annealing temperature is increased to 550 °C.
(4) The φ0.020 mm Ag-8.5Au-3.5Pd alloy wire will shake seriously in the annealing tube if the tension is small (1.5–2.0 g), which would cause burns on the wire surface. When the tension is large (3.5 g), corrugated defects would appear on the surface of the alloy wire. The alloy wire has a high-quality surface when the tension range is 2.5–3.0 g.

## Figures and Tables

**Table 1:** Wire drawing die dimensions.

Step	Range of Dies Hole Size (mm)	Dies Sleeve Size (mm)		
		External Diameter	Thickness	Tolerance
Rough drawing	5.210~7.628	42	27	+0, −0.1
	1.513~4.798	42	27	+0, −0.1
Medium drawing	0.5480~1.4138	25	8	+0, −0.1
	0.1985~0.5121	25	8	+0, −0.1
Fine drawing 1	0.11380–0.19170	25	6	+0, −0.02
Fine drawing 2	0.06525–0.010991	25	6	+0, −0.02
Fine drawing 3	0.03874–0.06032	25	6	+0, −0.02
Ultra-fine drawing 1	0.03035~0.03780	25	6	+0, −0.02
Ultra-fine drawing 2	bellow 0.03	25	6	+0, −0.02
Ultra-fine drawing 3	bellow 0.03	25	6	+0, −0.02

**Table 2:** Wire drawing die dimensions.

Step	Range of Dies Hole Size (mm)	Aperture Tolerance (mm)	Roundness (mm)	Entry Angle (°)	Bearing Length (%D)	Exit Zone Angle (°)
Rough drawing	5.210~7.628	+0, −0.004	0.004	15° ± 2°	30 ± 10	15° ± 5°
	1.513~4.798	+0, −0.003	0.003	15° ± 2°	30 ± 10	15° ± 5°
Medium drawing	0.5480~1.4138	+0, −0.002	0.001	15° ± 2°	20~30	15° ± 5°
	0.1985~0.5121	+0, −0.001	0.001	15° ± 2°	20~40	15° ± 5°
Fine drawing 1	0.11380~0.19170	+0, −0.0005	0.0005	13° ± 2°	50 ± 10	15° ± 5°
Fine drawing 2	0.06525~0.010991	+0, −0.0003	0.0003	13° ± 2°	50 ± 10	15° ± 5°
Fine drawing 3	0.03874~0.06032	+0, −0.0002	0.0002	13° ± 2°	50 ± 10	15° ± 5°
Ultra-fine drawing 1	0.03035~0.03780	+0, −0.0002	0.0002	13° ± 2°	50 ± 10	15° ± 5°
Ultra-fine drawing 2	bellow 0.03	+0, −0.0002	0.0002	13° ± 2°	50 ± 10	15° ± 5°
Ultra-fine drawing 3	bellow 0.03	+0, −0.0002	0.0002	13° ± 2°	50 ± 10	15° ± 5°

**Table 3:** Annealing process parameters of Ag-8.5Au-3.5Pd alloy wire.

Number	Temperature (°C)	Time (s)	Remarks
1	200	2.4	The annealing tube length is 2.0 m and the annealing speed is 50 m/min.
2	300	2.4	
3	400	2.4	
4	450	2.4	
5	500	2.4	
6	550	2.4	
